# Vorinostat Treatment of Gastric Cancer Cells Leads to ROS-Induced Cell Inhibition and a Complex Pattern of Molecular Alterations in Nrf2-Dependent Genes

**DOI:** 10.3390/ph17081080

**Published:** 2024-08-16

**Authors:** Leoni Lorenz, Tamara Zenz, Denys Oliinyk, Florian Meier-Rosar, Robert Jenke, Achim Aigner, Thomas Büch

**Affiliations:** 1 Clinical Pharmacology, Rudolf-Boehm-Institute for Pharmacology and Toxicology, Medical Faculty, Leipzig University, 04107 Leipzig, Germanytamara.zenz@medizin.uni-leipzig.de (T.Z.); robert.jenke@medizin.uni-leipzig.de (R.J.); 2 Functional Proteomics, Research Center Lobeda, Jena University Hospital, 07747 Jena, Germany; denys.oliinyk@med.uni-jena.de (D.O.); florian.meier-rosar@med.uni-jena.de (F.M.-R.); 3 Comprehensive Cancer Center Central Germany (CCCG), 04103 Leipzig, Germany; 4 Comprehensive Cancer Center Central Germany (CCCG), 07743 Jena, Germany; 5 University Cancer Center Leipzig (UCCL), University Hospital Leipzig, 04103 Leipzig, Germany

**Keywords:** histone deacetylase inhibitors, vorinostat, reactive oxygen species, Nrf2 signaling

## Abstract

Histone deacetylase inhibitors (HDACi) show high antineoplastic potential in preclinical studies in various solid tumors, including gastric carcinoma; however, their use in clinical studies has not yet yielded convincing efficacies. Thus, further studies on cellular/molecular effects of HDACi are needed, for improving clinical efficacy and identifying suitable combination partners. Here, we investigated the role of oxidative stress in gastric cancer cells upon treatment with HDACi. A particular focus was laid on the role of the Nrf2 pathway, which can mediate resistance to cell-inhibitory effects of reactive oxidative species (ROS). Using fluorescence-based ROS sensors, oxidative stress was measured in human gastric cancer cell lines. Activation of the Nrf2 pathway was monitored in luciferase reporter assays as well as by mRNA and proteomic expression analyses of Nrf2 regulators and Nrf2-induced genes. Furthermore, the effects of ROS scavenger N-acetyl-L-cysteine (NAC) and Nrf2-knockdown on HDACi-dependent antiproliferative effects were investigated in colorimetric formazan-based and clonogenic survival assays. HDACi treatment led to increased oxidative stress levels and consequently, treatment with NAC reduced cytotoxicity of HDACi. In addition, vorinostat treatment stimulated expression of a luciferase reporter under the control of an antioxidative response element, indicating activation of the Nrf2 system. This Nrf2 activation was only partially reversible by treatment with NAC, suggesting ROS independent pathways to contribute to HDACi-promoted Nrf2 activation. In line with its cytoprotective role, Nrf2 knockdown led to a sensitization against HDACi. Accordingly, the expression of antioxidant and detoxifying Nrf2 target genes was upregulated upon HDACi treatment. In conclusion, oxidative stress induction upon HDAC inhibition contributes to the antitumor effects of HDAC inhibitors, and activation of Nrf2 represents a potentially important adaptive response of gastric cancer cells in this context.

## 1. Introduction

Epigenetic alterations represent important and sometimes very early events in tumorigenesis. Thus, dysregulation at the epigenetic level is considered one of the hallmarks of cancer [1,2]. Consequently, anticancer therapeutics with epigenetic targets (“epidrugs”) have received increasing attention [3], also encouraging research into new treatment approaches in the case of gastric carcinoma [4]. The identification of a large number of epigenetic changes in this tumor entity may well provide the basis for novel strategies in therapeutic intervention [4]. For example, an association between altered expression and disease prognosis/progression has been described for a whole series of histone deacetylase (HDAC) subtypes [5]. Accordingly, HDAC inhibitors (HDACi) have demonstrated high antineoplastic potential in preclinical studies in gastric carcinoma [6,7,8]. However, the use of HDACi in clinical studies has not yet yielded convincing efficacies [9,10], thus highlighting the need for identifying suitable combination partners and for further studying cellular/molecular effects of HDACi in greater detail in order to improve clinical efficacy [11]. Rational combination therapies should be based on the mechanistic understanding of the antineoplastic effects of HDACi in gastric cancer, also taking possible resistance factors into account. This is further complicated by the fact that HDACi exert complex effects through the downregulation of various oncogene pathways or the upregulation of tumor-suppressor genes and the induction of DNA damage [11]. An important endpoint of these effects is the induction of cell cycle arrest. This is usually accompanied by the p53-dependent or -independent induction of p21 (CDKN1A) [11], which represents a master regulator of cell cycle regulation.

An important aspect in the mechanisms of action of HDACi is their multi-dimensional impact on oxidative stress. In principle, HDACi treatment can be associated with increased formation of reactive oxygen species (ROS) [11]. However, this may be either causally linked to the antitumor effect of HDACi or represent a mere epiphenomenon; in other words, increased oxidative stress after HDACi treatment may be the cause or the consequence of cytotoxic HDACi effects. Of note, we were recently able to show that HDACi treatment of gastric carcinoma cells is associated with the activation of ferroptosis-regulating pathways [12]. In fact, HDACi treatment of gastric cancer cells led to the parallel induction of pro-ferroptotic and downregulation of anti-ferroptotic regulators [12]. Ferroptosis represents a variant of programmed cell death, which is characterized by oxidative membrane damage leading to a loss of membrane integrity with release of pro-inflammatory stimuli. The fact that ROS formation is a necessary component of ferroptosis-mediated cell death indicates a causal role of oxidative stress in the tumor-inhibiting effect of HDACi in gastric carcinoma. In contrast, HDACi have also been shown to stimulate antioxidant defense mechanisms, which in turn may lead to reduced tumor cytotoxic effects of HDACi as well as impaired antineoplastic activity of other, co-administered chemotherapeutic agents. In this context, the transcription factor Nrf2 is of particular importance by stimulating the expression of a whole range of antioxidant, cytoprotective, and detoxifying factors. Thus, Nrf2 contributes to cellular resistance to oxidative stress and a number of cell-damaging agents [13,14].

As in the case of ROS induction vs. antioxidative defense (see above), quite opposing effects of HDACi on the Nrf2 system have been described, depending on the respective cellular system. For example, one study using a substance screen with MCF-7 breast cancer cells revealed that different HDACi, including vorinostat, increased Nrf2 signaling [15]. Indeed, this effect impaired the antineoplastic efficacy of co-administered cytotoxic agents [15]. Likewise, the HDACi trichostatin and vorinostat stimulated autophagic survival pathways in hepatocellular carcinoma cells (Huh-7) or in MGC80-3 cells (somatic hybrid gastric carcinoma cells with HeLa cells) via a Nrf2-dependent mechanism [16]. This HDACi-mediated Nrf2 activation, associated with increased cellular resistance toward HDACi and other cytotoxic compounds, suggests that the simultaneous inhibition of Nrf2 could lead to increased HDACi sensitivity and counteract HDACi-dependent resistance toward other cytostatic drugs.

In contrast, however, other studies demonstrated HDACi-mediated inhibition of the Nrf2 system and a concomitant reduction of pro-survival factors. For example, HDAC2 knockdown or treatment with the HDACi trichostatin prevented the activation of Nrf2 by oxidative stress in the bronchial epithelium [17]. Accordingly, vorinostat acted synergistically with tyrosine kinase inhibitors [18] by inhibiting Nrf2 signaling in non-small-cell lung cancer cells. Likewise, increased oxidative stress after HDACi treatment of sarcoma [19] or myeloid leukemia cells [20] was also associated with an inhibition of the Nrf2 system. Interestingly, inhibition of Nrf2 upon vorinostat treatment has also been demonstrated in colorectal cancer cells, this in turn leading to their sensitization toward platinum-containing cytostatic drugs [21].

The role of oxidative stress and a possible modulation of the Nrf2 system by HDACi in gastric cancer is still poorly understood. For this reason, the present study tested if and to what extent HDACi treatment affects oxidative stress in gastric cancer cells and whether antioxidant treatment reduces tumor-inhibitory activity of HDACi. On the molecular level, this included effects of HDACi on Nrf2 and the expression of Nrf2-dependent genes.

Based on our above-mentioned previous findings [12] on the HDACi effects on ferroptosis pathways, we pursued the hypothesis that in the case of gastric carcinoma an induction of oxidative stress might contribute significantly to the antitumor effect of HDACi. A possible induction of Nrf2 signaling pathways would be a conceivable adaptive response in this scenario and lead to resistance to HDACi. In this sense, this would provide the basis for future strategies toward the enhancement of HDACi-mediated antineoplastic activity.

## 2. Results

### 2.1. Vorinostat Induces Reactive Oxygen Species (ROS) Increase in Gastric Carcinoma Cells

**Figure 1:**
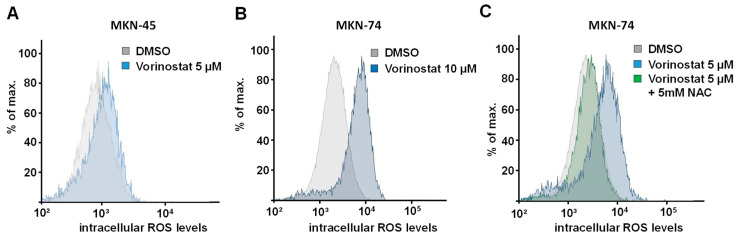
Effect of 48 h vorinostat treatment, alone or in combination with N-acetyl-L-cysteine (NAC), on oxidative stress in (**A**) MKN-45 and (**B**,**C**) MKN-74 cells. The formation of reactive oxidative species (ROS) was determined by the increase in fluorescence of the ROS-sensitive reagent CellRox Deep Red. A right shift of the fluorescence distribution indicates increased ROS formation. Representative FACS analyses of three independent experiments are shown, with each experiment being performed in duplicates.

In the first step, the vorinostat-mediated induction of oxidative stress was investigated in a panel of gastric carcinoma cells. CellROX DeepRed was used as a fluorogenic sensor for reactive oxygen species (ROS). After 48 h treatment of MKN-45 cells with 5 µM vorinostat, flow cytometry showed an increase in fluorescence intensity, indicating increased intracellular ROS levels (Figure 1A). An even more profound ROS increase was seen in MKN-74 cells treated with 10 µM vorinostat (Figure 1B). The direct comparison with 5 µM vorinostat treatment revealed little dose-dependence in this and other cell lines (compare Figure 1B,C). To avoid our results being influenced by other cytotoxic vorinostat effects, the lower dosages were selected for further experiments.

Of note, the differences in the extent of the vorinostat-mediated rightward shift (=ROS production) in MKN-74 cells (Figure 1B,C) vs. MKN-45 (Figure 1A) or Hs746T cells (Supplementary Figure S1A) show that cell context-dependent differences exist between different gastric cancer cell lines. These differences could lead to a different weighting of ROS-dependent and ROS-independent antiproliferative effects of HDACi depending on the cellular background. The addition of N-acetyl-L-cysteine (NAC) partially reverted the observed ROS increases (Figure 1C). This also allowed for dissecting ROS-associated vorinostat effects from other tumor cell inhibitory effects of the drug (see below).

A vorinostat-mediated induction of ROS was also observed in Hs746T cells (Supplementary Figure S1A). Notably, similar effects were also obtained when treating the same cell line with the HDACi entinostat, with 3 µM already leading to a profound right-shift (Supplementary Figure S1B). This also confirms that the pro-oxidative effects were related to the general HDAC inhibitory potential of the tested compounds rather than any other compound-specific effect of a given drug.

### 2.2. Tumor Cell Inhibition upon Vorinostat-Mediated ROS Increase

**Figure 2:**
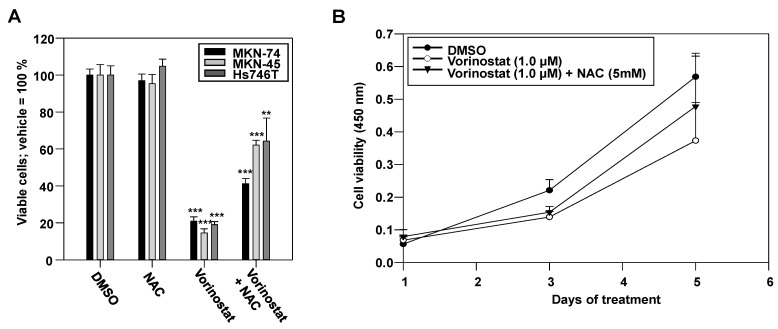
Vorinostat-dependent growth inhibition in MKN-74, MKN-45, and Hs746T cells. (**A**) Determination of viable cells after 48 h treatment with 5 µM vorinostat and/or 5 mM NAC as compared to vehicle (DMSO) control. (**B**) Effects of a lower vorinostat concentration (1 µM) on cell growth over 5 days and its modulation by NAC in MKN-45 cells. Of note, 25,000 cells in a 12-well format were used for the short-term incubation (**A**) and 750 cells in a 96-well format were used for the long-term incubation (**B**), which also affects the susceptibility of the cells to the treatment. Cell viability was determined using the formazan-based CCK8 assay. Each bar or point in the diagram represents the mean of at least three independent experiments + SEM. **, *p* < 0.01 and ***, *p* < 0.001.

Next, we analyzed if the observed pro-oxidative effect contributes to tumor cell-inhibition upon vorinostat treatment. As expected, proliferation assays revealed profound anti-tumor effects in all tested gastric carcinoma cell lines (Figure 2A,B). Although MKN-74 showed a more profound increase in intracellular ROS levels upon vorinostat treatment as compared to the other cell lines (Figure 1, Supplementary Figure S1), the sensitivities of the cell lines MKN-74, MKN-45, and Hs746T toward 5 µM vorinostat were comparable (Figure 2A).

Notably, the addition of N-acetyl-L-cysteine (NAC) partially rescued the cells from the vorinostat-mediated inhibition (Figure 1A,B), indicating that ROS induction is a relevant cytostatic effect of the drug. Despite the very profound vorinostat-induced increase of ROS in MKN-74 cells and the almost complete rescue under NAC treatment (see Figure 1B,C), the addition of NAC was less efficient in alleviating vorinostat effects in this cell line as compared to MKN-45 and Hs746T cells (Figure 2A). This may indicate cell line-dependent differences in the relative contribution of oxidative stress in vorinostat-dependent antitumor effects. Taken together, these data establish ROS induction as one underlying mechanism of vorinostat-mediated cell inhibition.

### 2.3. ROS-Dependent Induction of Antioxidant Response Element (ARE)-Regulated Luciferase Reporter upon Vorinostat Treatment

The transcription factor Nrf2 (Nuclear factor erythroid 2-related factor 2) is a pivotal regulator of cellular defense mechanisms against oxidative stress. The activation of the Nrf2 system induces antioxidant response element (ARE)-driven transcription. Thus, based on our findings of vorinostat-mediated ROS induction suggesting adaptive responses to be induced in the tumor cells, we next employed a luciferase expression plasmid under the control of ARE as a reporter construct for monitoring Nrf2-promoted gene expression.

**Figure 3:**
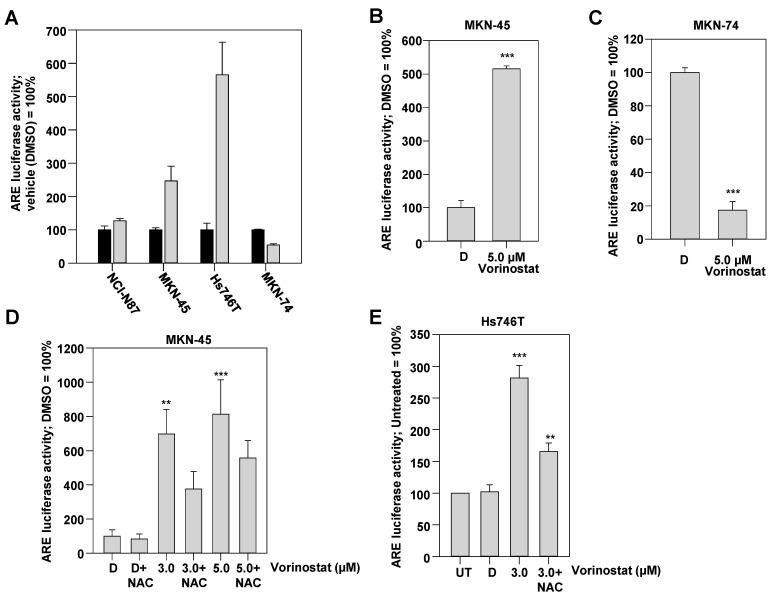
Determination of Nrf2 activity upon vorinostat treatment, alone or in combination with N-acetyl-L-cysteine (NAC), via luciferase reporter assay. (**A**) Treatment of a panel of cell lines with 3 µM vorinostat (gray bars) or vehicle (DMSO) (black bars) for 48 h led to vorinostat-induced increase of luciferase activity, indicating Nrf2 activation in most cell lines. Data represent the mean of luciferase measurements performed in duplicate. Additional experiments were performed in (**B**) MKN-45 and (**C**) MKN-74 cells treated with 5 µM vorinostat for 48 h or vehicle DMSO (“D”), showing opposite vorinostat effects on luciferase activity. Furthermore, the effect of NAC on vorinostat-mediated increase in luciferase activity was tested in (**D**) MKN-45 and (**E**) Hs746T cells. Graphs in (**B**–**E**) show mean values of at least three independent experiments + SEM. **, *p* < 0.01 and ***, *p* < 0.001.

A first screen in a panel of gastric carcinoma cell lines revealed in most cases a slight (NCI-N87) or profound > twofold (MKN-45, Hs746T) activation of the Nrf2 reporter (Figure 3A). Inhibition was only seen in the cell line MKN-74 (Figure 3A). These findings of reporter gene activation in MKN-45 cells and the opposite behaviour in MKN-74 cells was confirmed in follow-up experiments (Figure 3B,C), underlining the cell-dependency and complexity of the vorinostat effects on the Nrf2 system.

To further study the relationship between vorinostat treatment and the activation of Nrf2, the influence of NAC on the vorinostat-driven activation of Nrf2 was investigated as well. In MKN-45 as well as Hs746T cells, NAC partially attenuated the stimulation of the Nrf2 reporter upon 3 µM vorinostat treatment (Figure 3D,E). Again, little dose-dependency of vorinostat effects were observed, with an only minor increase of Nrf2 activation upon treatment with 5 µM instead of 3 µM vorinostat. However, the rescuing effect of NAC was less efficient at the higher concentration (Figure 3D). This incomplete inhibitory effect of NAC on HDACi-mediated activation of Nrf2 is consistent with the notion that HDACi-dependent but ROS-independent pathways may contribute to Nrf2 stimulation as well. Indeed, the analysis of potential Nrf2 regulators provided evidence for the presence of such ROS-independent effects (see below).

### 2.4. Nrf2 Knockdown Leads to a Cell Line-Dependent Enhancement of Tumor Cell Inhibition upon Vorinostat Treatment

**Figure 4:**
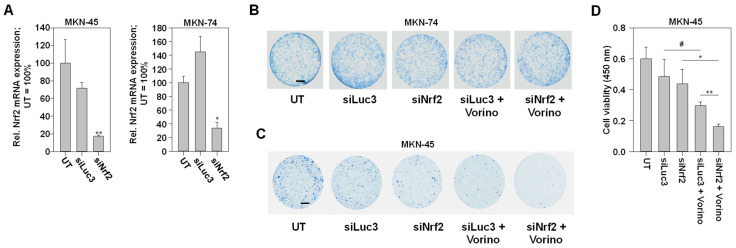
(**A**) RNAi-mediated knockdown of Nrf2 in MKN-45 and MKN-74 cells, as determined on mRNA level. Cells were transfected with Nrf2-specific siRNA (siNrf2) or control siRNA (siLuc3), and Nrf2 expression was evaluated 48 h after transfection. (**B**,**C**) Effects of RNAi-mediated Nrf2 knockdown (siNrf2) on vorinostat toxicity, compared to a transfection with an unspecific control siRNA (siLuc3) or untreated cells (UT) in colony-formation assays. Scale bars indicate 5 mm. Viable colonies of (**B**) MKN-74 cells and (**C**) MKN-45 cells were stained with methylene blue. Representative images of three independent experiments are shown. (**D**) Effects of long-term vorinostat treatment (1 µM) and its modulation by Nrf2 knockdown (siNrf2) versus control siRNA (siLuc3), as determined in a formazan-based CCK8 assay for viable cells. Cells were treated for 5 days as indicated. Shown are mean values of three independent experiments + SEM. #, *p* > 0.05; *, *p* < 0.05 and **, *p* < 0.01.

Considering the role of Nrf2 as a cytoprotective factor, we next investigated the influence of Nrf2 knockdown on the cytotoxicity of vorinostat in MKN-45 and MKN-74 cells. We selected these two cell lines due to the above-shown opposite vorinostat effects on Nrf2 (MKN-74: inhibition, MKN-45: activation). In colony formation assays, clonogenic cell survival of MKN-74 cells was unaffected by Nrf2 knockdown. Likewise, no effect on vorinostat cytotoxicity was observed (Figure 4B). In contrast, siNrf2 transfection of MKN-45 cells led to a reduction in colony formation and survival. Treatment with vorinostat yielded profound inhibition of clonogenic cell survival as well. Notably, however, the combination of both led to an almost complete abolishment of viable colonies (Figure 4C). A classical 2D cell viability assay showed comparable results. Again, the Nrf2 knockdown yielded a slight and the vorinostat treatment a somewhat more profound reduction in the number of viable cells. The most profound effects were again observed in the combined siNrf2 + vorinostat treatment (Figure 4D).

### 2.5. Vorinostat-Mediated Alterations in Expression Levels of Nrf2-Dependent Genes

To further elucidate the potentially multi-dimensional effects of HDACi on the Nrf2 system, we next investigated the influence of vorinostat on Nrf2 target genes in the cell lines MKN-45 and Hs746T, showing activation of Nrf2 by vorinostat, and in the cell line MKN-74, where a vorinostat-mediated Nrf2 inhibition had been observed. For this purpose, we chose established NRF2-regulated genes that have been already described as particularly important in the cancer context [22].

**Figure 5:**
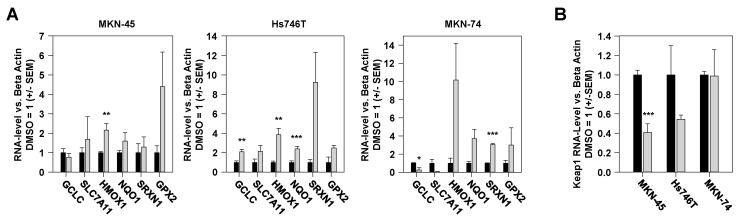
(**A**) RT-qPCR analysis of mRNA levels of Nrf2 target genes in MKN-45, Hs746T, and MKN-74 cells, treated with 3 µM vorinostat (grey) vs. DMSO (black) for 48 h. (**B**) Vorinostat effects on the mRNA levels of the Nrf2 regulator Keap1 in the three cell lines. Expression levels of the respective gene were normalized to beta-actin as the housekeeping gene, and the relative expression levels of vehicle (DMSO)-treated cells were set to 1. Mean values of three independent experiments + SEM are shown. *, *p* < 0.05; **, *p* < 0.01 and ***, *p* < 0.001.

Already on the mRNA level, a complex pattern of molecular alterations was observed. In all three gastric carcinoma cell lines, vorinostat led to increased mRNA levels of certain genes described as typical Nrf2 target genes (Figure 5A). Besides these more general effects, a distinct pattern was observed in each of the three cell lines. In MKN-45 cells, the induction of glutathione peroxidase 2 (GPX2) was particularly striking, while in Hs746T cells dominated the upregulation of sulfiredoxin 1 (SRXN1), and in MKN-74 cells the increased expression of heme oxygenase 1 (HMOX1), respectively (Figure 5A). The latter finding was surprising at first glance considering the above observation of vorinostat-mediated inhibition of Nrf2 in MKN-74 cells. However, it must be taken into account that HMOX1 can also be induced by numerous other regulators such as hypoxia inducible factor, NFκB, or AP-1 [23]. Of note, the downregulation of the Nrf2 target genes GCLC and SLC7A11 (Figure 5A) in MKN-74 cells was consistent with our previously observed Nrf2 inhibition in this cell line. In the other two cell lines, MKN-45 and Hs746T, vorinostat treatment also led to reduced mRNA levels of the negative Nrf2 regulator Keap1 (Figure 5B), which is in line with the observed upregulation of Nrf2.

**Figure 6:**
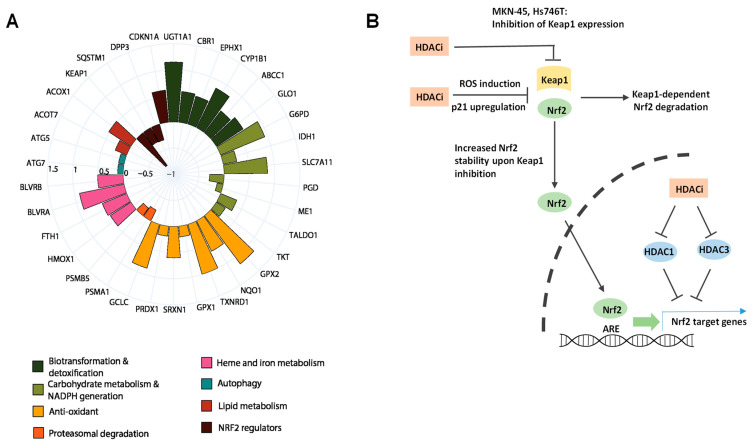
(**A**) Proteomics analyses of vorinostat vs. DMSO treated MKN-45 cells. Expression pattern alterations of various Nrf2 regulators such as Keap1, p21 (CDKN1A), p62 (SQSTM1), and DPP3, as well as of Nrf2 target genes, are shown. Lower panel: group association of altered genes to different biochemical/cellular functions. (**B**) Schematic overview of the complex and multimodal pattern of HDACi effects on Nrf2 levels and activity.

To confirm this also on the proteome level, we extracted protein abundances of Nrf2 regulators such as Keap1, p21 (CDKN1A), p62 (SQSTM1), and DPP3, and of Nrf2 target genes from our previous proteome-wide study of MKN-45 cells incubated with vorinostat [12] (Figure 6A). The expression of Keap1, which is the most important inhibitor of Nrf2, was clearly reduced by vorinostat, consistent with the above mRNA results. Conversely, p21 (CDKN1A), a partially ROS-independent activator of the Nrf2 system, showed a clear upregulation (Figure 6A). Taken together, this revealed a complex and multimodal pattern of HDACi effects on Nrf2 levels and activity (Figure 6B), which is also consistent with the different effects of HDACi in different cells. In particular, the vorinostat-induced Nrf2 activation may be mediated via ROS-dependent inhibition of the negative Nrf2 regulator Keap1 or via the partially ROS-independent positive Nrf2 regulator p21 (Figure 6B). In addition, downstream inhibition of HDAC1 and HDAC3 leading to a de-repression of Nrf2 target genes may be involved in the vorinostat-dependent activation of the Nrf2 system as well (Figure 6B).

With regard to Nrf2-regulated genes, a largely uniform upregulation was found on the protein level, particularly in the case of proteins with detoxifying or antioxidative function or regulators of iron metabolism (Figure 6A). These changes are again consistent with a cytoprotective function of Nrf2 in gastric cancer cells.

Thus, our findings highlight the possibility that HDACi may induce numerous proteins involved in cellular resistance against chemotherapeutics, which has to be taken into account when combining these agents with classic cytotoxic therapeutics.

## 3. Discussion

The present study demonstrates HDAC inhibition leading to increased oxidative stress in gastric carcinoma cells. More importantly, vorinostat was found to activate the transcription factor Nrf2. This vorinostat-mediated Nrf2 activation can be considered as an adaptive response towards treatment with HDACi, contributing to vorinostat resistance. Accordingly, the RNAi-mediated downregulation of Nrf2 led to enhanced antitumor effects of vorinostat, which may indicate novel therapeutic strategies based on dual inhibition. The cell line MKN-74 represented the only exception, with vorinostat causing decreased Nrf2 activity. This highlights the interpatient heterogeneity in gastric carcinoma, which is also reflected by differences in established cell lines. In this context, it is interesting to note that treatment with the ROS scavenger NAC was less effective in reducing the cytotoxicity of vorinostat in MKN-74 cells as compared to the other gastric carcinoma cell lines. Since vorinostat-mediated residual Nrf2 activation was still present in the other cell lines even under NAC treatment, this might have stimulated survival pathways that were not active in MKN-74 cells. This could be a possible hypothesis for explaining cell line-dependent differences in vorinostat susceptibility under NAC.

The role of Nrf2 in HDACi resistance is consistent with its general function as a master regulator of cytoprotective factors [24]. In fact, Nrf2 has been shown to activate a broad range of antioxidative systems as a defense mechanism against reactive oxygen species [25]. In addition, drug efflux transporters and detoxifying phase II enzymes have been found upregulated as well [26]. In this respect, however, Nrf2 plays an ambiguous role in the etiopathogenesis of tumors: On the one hand, a functional or mutation-induced inhibition of Nrf2 signaling in premalignant cells can be the starting point for the accumulation of further DNA damage and thus promote progression toward malignant neoplasia. On the other hand, the constitutive overactivation of Nrf2 promotes cell survival and can therefore represent a selection advantage for tumor cells. It is therefore not surprising that in some cases defects of the Nrf2 system have been found in tumor cells, while in other cases an Nrf2 overactivity has been described [27]. The latter phenomenon may even result in an Nrf2 addiction of tumor cells, which is perhaps best characterized in bronchial carcinoma [28] but has also been found in several other entities, including gastrointestinal tumors [29].

Previous studies have already revealed the relevance of Nrf2 in particular in adenocarcinomas of the upper gastrointestinal tract (gastric carcinoma and oesophageal adenocarcinoma). The increased co-expression of Nrf2 and an Nrf2 activating protein, FAM117B, was found in the tumor tissue of patients with gastric carcinoma and was associated with a poorer prognosis [30]. Moreover, increased Nrf2 activity caused increased tumor growth and chemoresistance [30]. Interestingly, two typical cell-damaging events of the upper gastrointestinal tract have been described to modulate the Nrf2 system: extracellular acidification [31] and exposure to *Helicobacter pylori* [32,33]. Thus, the low pH environment in the upper gastrointestinal tract may contribute to increased therapy resistance of cancer cells via activation of Nrf2 [31]. In contrast, the more complex interaction of *Helicobacter pylori* with the Nrf2 system illustrates the dual function of Nrf2 in tumors and tumor progenitor cells. A short-term induction of Nrf2 by *Helicobacter pylori* infection was found to contribute to increased cell survival [32], whereas longer-term inhibition of the Nrf2 system after chronic infection has been associated with increased epithelial-mesenchymal transition (EMT) and has been proposed as a key factor in tumor progression in this entity [33].

Our findings in the present study introduce Nrf2 activation as a resistance factor against HDACi, suggesting the possibility of a stratified treatment approach: Tumors with defective Nrf2 regulation may show a higher sensitivity to HDACi than tumors with normal Nrf2 function, while, vice versa, cancer cells with constitutively active Nrf2 may have a lower susceptibility to HDACi, thus requiring Nrf2 inhibition or knockdown for enhancing HDACi efficacy. However, one obstacle in this context is the fact that Nrf2 is difficult to target due to its complex regulation and the lack of classical binding pockets for antagonists. Nevertheless, a number of compounds have been identified, which have shown promising activity, e.g., trigonellin, brusatol, or halofuginone [13,34]. However, small molecule inhibitors of Nrf2 developed to date often act indirectly by inhibiting Nrf2-activating pathways or show limitations in their duration of action or potency/selectivity toward Nrf2 [34,35]. For this reason, molecular glues or approaches for the specific downregulation of Nrf2 via proteolysis targeting chimera (PROTACs) are an interesting alternative to classical small molecule inhibitors [35]. Alternatively, siRNA-mediated knockdown approaches are promising as well, also bearing in mind that in other pathologies first siRNA drugs have succeeded in clinical translation, despite still existing challenges [36].

Furthermore, the differential response of gastric cancer cells toward HDACi treatment emphasizes the complexity of Nrf2 regulation. The canonical activation pathway for Nrf2 starts with increased oxidative stress [37]. This causes inhibition of Keap1 through the oxidation of critical cysteine residues. In the absence of oxidative stress, Keap1 constitutively inhibits Nrf2 through direct protein-protein interaction with subsequent ubiquitination-driven Nrf2 degradation [37]. Oxidative inhibition of Keap1 thus leads to increased Nrf2 activity. However, non-canonical, partially redox-independent signal transduction pathways leading to Nrf2 stimulation exist as well. For example, p21, SQSTM1, or DPP3 inhibit the interaction of Keap1 with Nrf2, and by this means can also mediate a de-repression of this transcription factor [38].

The data presented here indicate that at least the CDKN1a (p21)-mediated Nrf2 activation could play a role in the context of non-canonical (i.e., ROS-independent) stimulation. Albeit in our proteome analyses revealed a slight downregulation of the Nrf2 activators DPP3 and SQSTM1, we found a clear, vorinostat-mediated induction of CDKN1a that has been described for HDACi in other contexts as well [39,40]. This alternative, CDKN1a-dependent activation pathway would also explain why the ROS scavenger N-acetyl-L-cysteine was not able to fully block and revert the vorinostat-mediated activation of Nrf2. Another Keap1-dependent regulatory mechanism of Nrf2 activity is the control of Keap1 expression. In fact, Keap1 expression is modulated at different levels (i.e., on the transcriptional, translational, and post-translational level) [41]. For example, Keap1 inhibition on the mRNA level has been described to occur in breast cancer following HDACi-dependent upregulation of micoRNAs (e.g., miR-200) [42]. Concomitantly, in the gastric carcinoma cells studied here, we were able to detect a vorinostat-mediated inhibition of Keap1 on both the protein and the mRNA level, suggesting effects on the transcript to play a role as well. Notably, it has also been shown that the Nrf2 system can be modulated through the direct transcriptional regulation of Nrf2 itself [43], adding further complexity to this pathway.

Finally, our results also indicate that any therapy based on HDACi may also influence the susceptibility to other drugs, in particular chemotherapeutics, via modulation of the Nrf2 system. Depending on the initial level of Nrf2 activity and the cumulative effects of HDACi on the Nrf2 system, however, this can lead to quite different effects among individuals. Based on the findings presented here, the HDACi-mediated influence on the therapeutic efficacies of possible combination partners in gastric carcinoma emerges as a promising avenue for defining novel concepts in gastric cancer therapy.

With regard to possible limitations of the present study, it should be kept in mind that our findings are based on classical cell culture experiments with gastric carcinoma cells. This allowed us to characterize basic mechanisms of HDACi-mediated tumor inhibition and to study the endogenous adaptive responses in the tumor cell. However, this model does not represent some parameters such as intratumoral heterogeneity or effects of stromal cells, which are important from a translational perspective. In order to address these aspects, the results obtained here should therefore be verified in more complex ex vivo models such as tumor slice cultures [44] or in vivo, for example in murine tumor models [45]. Moreover, we will make use of already established gastric cancer patient-derived xenograft models [12] for ongoing in vivo studies regarding therapeutic interventions in the context of the Nrf2 system. Additionally, off-target interactions of HDACi should also be kept in mind. Here, further analyses of the proteomics data may give valuable information.

## 4. Materials and Methods

### 4.1. Reagents

Acetonitrile (ACN), ≤30 ppm H₂O, was from Merck-Millipore (Darmstadt, Germany). Dimethylsulfoxide (DMSO), ≥99.8% p. a., tris-(hydroxymethyl)-amino-methane hydrochloride (Tris-HCl), ≥99% p. a., and tris(2-carboxyethyl)phosphine, >98% were obtained from Carl Roth (Karlsruhe, Germany). 2-chloroacetamide, 98%, and trifluoroacetic acid (TFA), 99%, were from Thermo Scientific Chemicals (Schwerte, Germany). N-acetyl-L-cysteine (NAC), 99.95%, was from Selleckchem (Houston, TX, USA).

### 4.2. Cell Culture

The cell lines MKN-45 (RRID:CVCL_0434), MKN-74 (RRID:CVCL_2791), Hs746T (RRID:CVCL_0333), and NCI-N87 (RRID:CVCL_1603) were obtained from the American Type Culture Collection (ATCC, Manassas, VA, USA). All cell lines are human gastric adenocarcinoma cells; however, they show distinct features and differences regarding critical oncogene alterations. MKN-45 cells carry a genomic amplification of the MET oncogene, while Hs746T are characterized by MET amplification and an exon 14 deletion in this gene [46]. NCI-N87 show an amplification of HER2 (ErbB2) [47]. In contrast, MKN-74 have no known alterations of classic oncogenic receptor tyrosine kinases. Cells were cultured under standard conditions (37 °C, 5% CO_2_) in DMEM medium supplemented with 4 mM L-glutamine, 4500 mg/L glucose, 1 mM sodium pyruvate, and 1500 mg/L sodium bicarbonate (Hs746T cells) or RPMI-1640 medium (MKN-45, MKN-74, and NCI-N87 cells). Both media as well as medium supplements and trypsin/EDTA were from Sigma-Aldrich (Taufkirchen, Germany) and were supplemented with 10% fetal calf serum (SERANA, Pessin, Germany). All cell lines were authenticated by short repeat tandem profiling during the last 3 years and were cultured for less than 15 passages. All cell cultures were regularly screened for mycoplasma contamination using a PCR Mycoplasma detection kit (Venor GeM Classic, minerva biolabs, Berlin, Germany).

### 4.3. Cell Treatment and Transfection

**Figure 7:**
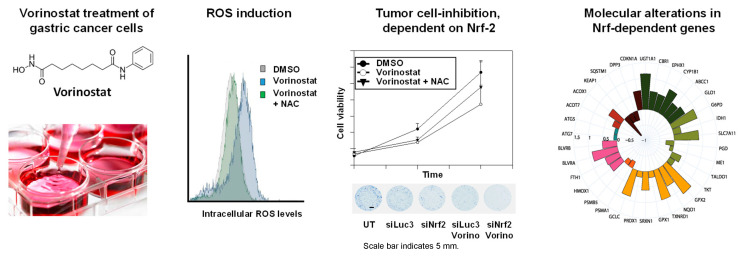
Schematic depiction of the workflow of cell-treatment analysis.

Treatment of cells with vorinostat (MedChemExpress, Monmouth Junction, NJ, USA) or N-acetyl-L-cysteine (NAC) in vitro was performed 24 h after seeding. A scheme of the workflow of cell treatment and analysis is shown in Figure 7. Vorinostat concentrations were chosen according to our previous findings, indicating half maximal toxicity in a range between 3.2 µM and 10.1 µM [48]. The selected NAC concentration was based of literature findings [49]. Solvent (DMSO)-treated cells were used as negative control. siRNA against Nrf2 and luciferase (negative control) were obtained from Eurofins MWG Operon (Ebersberg, Germany). The sequence of Nrf2 siRNA was 5′-GAAGCCAGAUGUUAAGAAAdTdT-3′ and 5′-UUUCUUAACAUCUGGCUUCdTdT-3′ for the sense and antisense strand, respectively. The sequence for the control siRNA (siLuc3) was 5′-CUUACGCUGAGUACUUCGAdTdT-3′ (sense strand) and 5′-UCGAAGUACUCAGCGUAAGdTdT-3′ (antisense strand). Transfection was carried out using INTERFERin (Polyplus, Illkirch, France), with 0.5 μL INTERFERin/pmol siRNA. For the transfections, 10 nM siRNA was used.

### 4.4. Fluorescence-Based Determination of Reactive Oxygen Species (ROS)

The CellROX Deep Red reagent from Thermo Scientific (Waltham, MA, USA) was used for detection of HDACi-dependent oxidative stress. In oxidized form, this reagent shows a distinct fluorescence with an emission peak at a wavelength of 665 nm. The cells were seeded on day 1 in 12-well plates at 50,000 cells/well, 24 h before treatment start. After another 48 h, the medium was replaced for medium containing 2.5 µM CellROX Deep Red reagent and the cells were incubated for 30 min at 37 °C in an incubator. After washing three times with phosphate-buffered saline (PBS), the cells were detached with trypsin/EDTA and the cell suspension was centrifuged at 700× *g* for 5 min. The pellet was resuspended in 500 µL PBS, and the cell suspension was then measured at an excitation wavelength of 640 nm in an Attune Acoustic Focusing Cytometer with Attune Cytometric Software Version 2.1.0 (Waltham, MA, USA) according to the manufacturer’s instructions and as previously described [50].

### 4.5. ARE Luciferase Reporter Assay

2–3 × 10^4^ cells per treatment condition were seeded into 24-well multititer plates. After overnight incubation, cells were transfected with 250 ng ARE Reporter construct (Promega, Walldorf, Germany). Twenty-four hours after transfection, the cells were treated with the respective agents in normal growth medium and allowed to grow for a further 24 h. The activity of the reporter assay was determined by Luciferase Dual Glo (Promega) according to the manufacturer’s protocol. In brief, the culture medium was aspirated, cells were washed with PBS, and 100 µL cell lysis buffer (Promega) were added per well. After 20 min incubation at room temperature, 10 µL lysate was mixed with 25 µL D-luciferin enzyme mix (Promega). Light emission was determined using a luminometer FB12 (Berthold Detection Systems, Bad Wildbad, Germany).

### 4.6. RNA Isolation and RT-qPCR Analyses

Depending on the cell line, 1.0–1.5 × 10⁵ cells were seeded per well of a six-well plate. The RNA Magic Reagent (Biobudget, Krefeld, Germany) was used for RNA extraction according to the manufacturer’s protocol. The Reverse Transcription of the total RNA was performed using the RevertAid RT Kit (Thermo Fisher Scientific, Waltham, MA, USA). The subsequent quantitative PCR was performed using the PerfeCTa^®^ SYBR^®^ Green FastMix^®^ ROX (QuantaBio, Hilden, Germany) in the StepOnePlus™ Real-Time System (Thermo Scientific, Darmstadt, Germany). The master mix was prepared according to the manufacturer’s protocol, and the qPCR was carried out under the following conditions: activation for 2 min at 95 °C, followed by 45 cycles of 10 s at 95 °C, 15 s at 55 °C, and 15 s at 72 °C, with recording of the fluorescence intensity at the end of each cycle. Primer sequences used for analyses are given in Supplementary Table S1. For PCR product analysis, the samples were incubated at 65 °C for 15 s and then heated up to 95 °C to obtain a melting curve. The housekeeping gene actin was used as a reference for normalization, since actin expression is not influenced by HDACi treatment [12]. For normalization, each sample was run with an actin-specific primer set and the target-specific primer set in parallel. Target levels were calculated by the formula 2^(CPHousekeeping gene − CPGene of interest)^ and normalized for untreated or vehicle (DMSO)-treated samples as described.

### 4.7. Proteomic Analyses of Nrf2 Target Gene Expression

To investigate protein abundance levels of Nrf2 target genes, we re-analyzed a subset of our previous analysis of proteome-wide responses to HDACi in different cell lines (de-posited to ProteomeXchange Consortium via the PRIDE [51] partner repository with the dataset identifier PXD050328) [12]. Briefly, three independent samples were prepared by treating MKN-45 cells with vehicle DMSO or 3 µM vorinostat, respectively, for 72 h, prior to adding lysis buffer (4% sodium deoxycholate, 100 mM Tris-HCl, pH 8.5), boiling for 10 min at 95 °C, sonication. The protein concentration was determined by bicinchoninic acid (BCA) assay (ThermoFisher, Darmstadt, Germany). Tris(2-carboxyethyl)phosphine and 2-chloroacetamide were added at final concentrations of 10 mM and 40 mM, respectively, and lysates were incubated for 5 min at 45 °C, prior to digestion overnight with trypsin and endoproteinase from *Lysobacter enzymogenes* (Lys-C) (Sigma-Aldrich, Taufkirchen, Germany) at an enzyme:protein ratio of 1:100 for both enzymes. Peptides were purified using styrenedivinylbenzene-reverse phase sulfonate (SDB-RPS) StageTips and reconstituted in mass spectrometry (MS) loading buffer (0.1% TFA/2% ACN) to a final concentration of 200 ng/µL.

Nanoflow reversed-phase liquid chromatography was performed on a Bruker Dal-tonics nanoElute system coupled to a trapped ion mobility—mass spectrometer (Bruker timsTOF HT) operated in dia-PASEF mode as previously described [52]. We sampled an ion mobility range from 1/K_0_ = 1.43 to 0.6 vs. cm^−2^ using 100 ms for both ion accumulation and mobility analysis. The collision energy was lowered linearly from 59 eV at 1/K_0_ = 1.4 vs. cm^−2^ to 20 eV at 1/K_0_ = 0.6 vs. cm^−2^. We calibrated trapped ion mobility spectrometry (TIMS) elution voltages to 1/K_0_ linearly using at least two out of three ions from Agilent ESI LC/MS tuning mix (*m*/*z*, 1/K_0_: 622.0289, 0.9848 vs. cm^−2^; 922.0097, 1.1895 vs. cm^−2^; and 1221.9906, 1.3820 vs. cm^−2^).

### 4.8. Proliferation Assay

We used formazan-based CCK8 assays to monitor cellular proliferation, relying on WST-8 (2-(2-methoxy-4-nitrophenyl)-3-(4-nitrophenyl)-5-(2,4-disulfophenyl)-2H-tetrazolium) as reagent, which represents a water-soluble variant of the well-established 3-(4,5-dimethyl-2-thiazolyl)-2,5-diphenyl-2H-tetrazolium bromide (MTT) reagent (WST = water soluble tetrazolium). To determine effects on proliferation after short-term treatment with 5 µM vorinostat, 2.5 × 10^4^ cells per well were seeded in 12-well plates, incubated for 6 h, and then treated for 48 h with the respective agents (vehicle (DMSO), vorinostat, or NAC). For evaluation of effects of 1 µM of vorinostat over 5 days, 750 cells per well were seeded in 96-well plates and cultivated overnight, prior to treatment start (day 0). At the defined time points, numbers of viable cells were determined using the CCK8 cell counting kit (Dojindo, Munich, Germany) according to the manufacturer’s protocol. Briefly, the medium was removed and CCK8 reagent, diluted 1:10 in medium, was added (50 µL per well in 96-well plates and 300 µL per well in 12-well plates). The CCK8 reagent was incubated for 30 min in 12-well and 1 h in 96-well experiments. Afterward, absorbance at 440 nm was determined in an ELISA plate reader, Multiskan FC (Thermo Scientific, Darmstadt, Germany). As a blank value, the absorbance was measured in a well without cells and subtracted from the other values.

### 4.9. Colony-Forming Assay

1 × 10⁵ cells were seeded per well of a 6-well plate, treated with siRNA or entinostat for 72 h as described above, then trypsinized and counted in a Neubauer counting chamber (Carl Roth). 1000 cells per sample were re-seeded in 6-wells in a total volume of 2 mL per well. After 8 days of growth, with a medium change every 72 h, the colonies were stained with methylene blue (1 mg/mL in 50% (*v*/*v*) ethanol). Pictures were taken of the 6-well plates with white, 3D-printed plastic inserts to enhance contrast.

### 4.10. Statistics

Statistical analyses were performed using SigmaPlot 14. Results are presented as mean ± standard error (SEM). Statistical significance of differences in all assays was assessed by two-sided Student’s *t*-test. A *p*-value of less than 0.05 was considered statistically significant, with *, *p* < 0.05; **, *p* < 0.01 and ***, and *p* < 0.001. Proteomics data analysis and visualization were performed using Perseus v1.6.15 (Max-Planck Institute of Biochemistry, Martinsried, Germany; https://maxquant.net/perseus/, accessed on 12 August 2024), custom scripts in R (4.0.1) and Python (3.8.8) with packages data.table (1.14.2), dplyr (1.0.7), ggplot2 (3.3.5), tidyR (1.1.14), patchwork (1.1.1), pandas (1.1.5), numpy (1.22.2), plotly (5.4.0), and scipy (1.7.3). If applicable, results were corrected for multiple hypothesis testing using the thresholds indicated in the results and figure legends.

## Data Availability

The mass spectrometry proteomics data are available from the ProteomeXchange Consortium via the PRIDE [51] partner repository with the dataset identifier PXD050328. All other data will be made available upon request.

## Supplementary Materials

The following supporting information can be downloaded at: https://www.mdpi.com/article/10.3390/ph17081080/s1, Figure S1: Effect of 48 h vorinostat (A) or entinostat (B) treatment on oxidative stress in Hs746T cells; Table S1: Primer sequences used in RT-qPCR analyses of Nrf2 target genes.
