# Supplementary material for: Vorinostat Treatment of Gastric Cancer Cells Leads to ROS-Induced Cell Inhibition and a Complex Pattern of Molecular Alterations in Nrf2-Dependent Genes
Source: Pharmaceuticals (Basel). 2024 Aug 16;17(8):1080. doi: 10.3390/ph17081080 (PMC11357633; doi:10.3390/ph17081080)
Supplement: Supplementary file 1 [file pharmaceuticals-17-01080-s001.zip › Lorenz et al - Suppl Figure S1.pptx]

## Slide 1
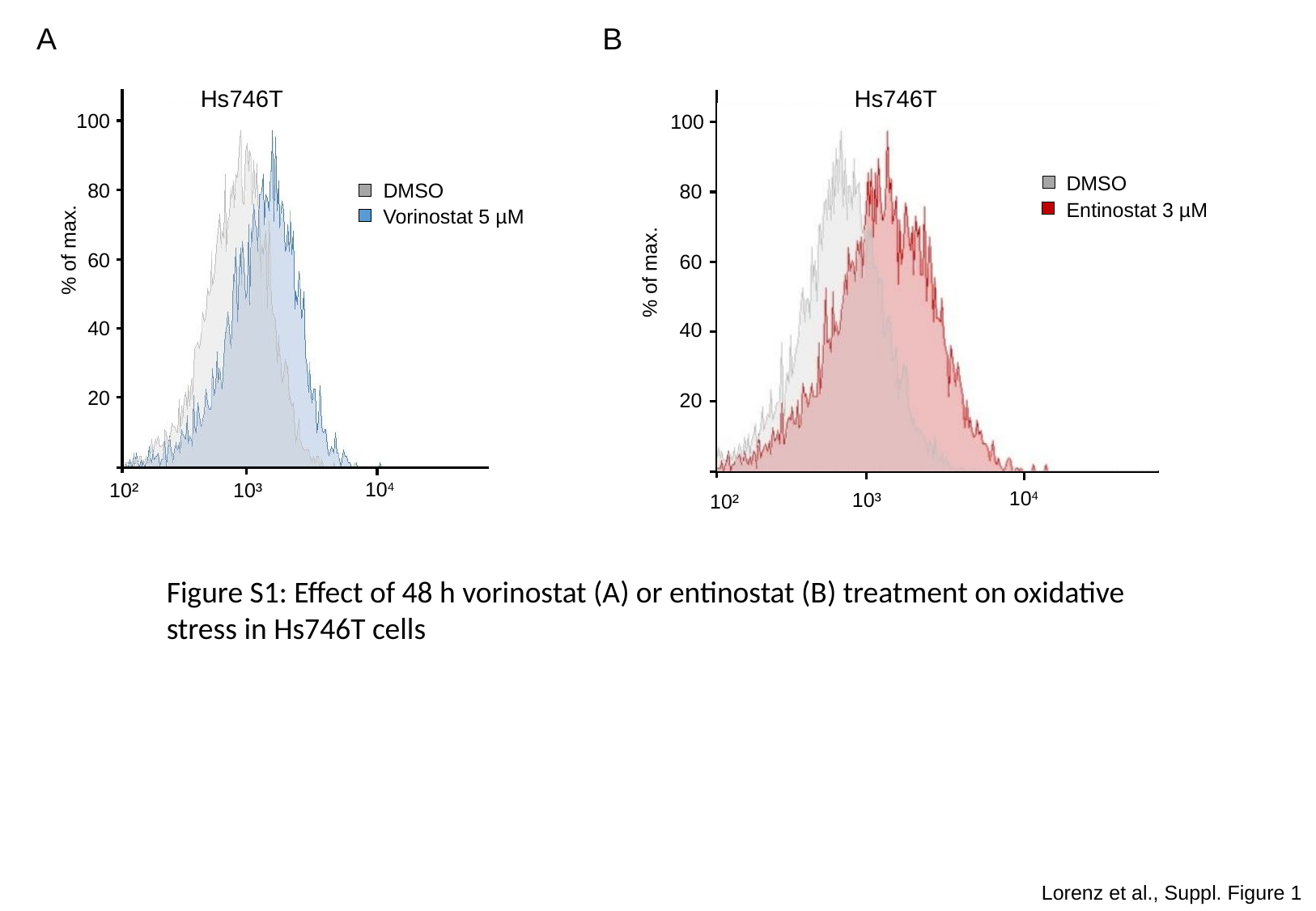

A
B
Hs746T
Hs746T
100
100
DMSO
Entinostat 3 µM
80
DMSO
Vorinostat 5 µM
80
% of max.
60
60
% of max.
40
40
20
20
104
10²
10³
104
10³
10²
Figure S1: Effect of 48 h vorinostat (A) or entinostat (B) treatment on oxidative stress in Hs746T cells
Lorenz et al., Suppl. Figure 1
