# Supplementary material for: Vorinostat Treatment of Gastric Cancer Cells Leads to ROS-Induced Cell Inhibition and a Complex Pattern of Molecular Alterations in Nrf2-Dependent Genes
Source: Pharmaceuticals (Basel). 2024 Aug 16;17(8):1080. doi: 10.3390/ph17081080 (PMC11357633; doi:10.3390/ph17081080)
Supplement: Supplementary file 1 [file pharmaceuticals-17-01080-s001.zip › Lorenz et al - Suppl. Table S1.pdf]

Suppl. Table S1: Primer sequences used in RT-qPCR analyses of Nrf2 target genes

**Gene**	**Sequence**
Actin for	CCAACCGCGAGAAGATGA
Actin rev	CCAGAGGCGTACAGGGATAG
GCLC for	TTG TCC TTT CCC CCT TCT CT
GCLC rev	CAA GGA CGT TCT CAA GTG GG
GPX2 for	GCC TCC TTA AAG TTG CCA TA
GPX2 rev	GCC CAG AGC TTA CCC A
HMOX1 for	GAG TGT AAG GAC CCA TCG GA
HMOX1 rev	GCC AGC AAC AAA GTG CAA G
Keap1 for	AAG AAC TCC TCT TGC TTG GC
Keap1 rev	CCA ACT TCG CTG AGC AGA TT
NQO1 for	GGG CAA GTC CAT CCC AAC TG
NQO1 rev	GCA AGT CAG GGA AGC CTG GA
NRF2 for	GCA TGA TGC CCA ATG TGA GA
NRF2 rev	TCC AAG CGG CTT GAA TGT TT
SRXN1 for	GCA GAG CCT CGT GGA CAC GAT
SRXN1 rev	ATG GTC TCT CGC TGC AGT TGC T
SLC7A11 for	TCC TGC TTT GGC TCC ATG AAC G
SLC7A11 rev	AGA GGA GTG TGC TTG CGG ACA T
